# Simplifying the Surgical Classification and Approach to the Posterolateral Skull Base and Jugular Foramen Using Anatomical Triangles

**DOI:** 10.7759/cureus.19638

**Published:** 2021-11-16

**Authors:** Jaafar Basma, Kara A Parikh, Nickalus R Khan, L. Madison Michael II, Jeffrey M Sorenson, Jon H Robertson

**Affiliations:** 1 Neurological Surgery, The University of Tennessee Health Science Center, Memphis, USA; 2 Laboratory, Medical Education Research Institute, Memphis, USA; 3 Neurosurgery, The University of Tennessee Health Science Center, Memphis, USA; 4 Neurological Surgery, Semmes-Murphey Clinic, Memphis, USA

**Keywords:** meningioma, glomus jugulare, hypoglossal schwannoma, distal cervical approach, hypoglossal canal, styloid process, infralabyrinthine approach, far lateral, jugular foramen

## Abstract

Introduction

Lesions of the jugular foramen (JF) and postero-lateral skull base are difficult to expose and exhibit complex neurovascular relationships. Given their rarity and the increasing use of radiosurgery, neurosurgeons are becoming less experienced with their surgical management. Anatomical factors are crucial in designing the approach to achieve a maximal safe resection.

Methods and methods

Six cadaveric heads (12 sides) were dissected via combined post-auricular infralabyrinthine and distal transcervical approach with additional anterior transstyloid and posterior far lateral exposures. Contiguous surgical triangles were measured, and contents were analyzed. Thirty-one patients (32 lesions) were treated surgically between 2000 and 2016 through different variations of the retro-auricular distal cervical transtemporal approaches.

Results

We anatomically reviewed the carotid, stylodigastric, jugular, condylar, suboccipital, deep condylar, mastoid, suprajugular, suprahypoglossal (infrajugular), and infrahypoglossal triangles. Tumors included glomus jugulare, lower cranial nerve schwannomas or neurofibromas, meningiomas, chondrosarcoma, adenocystic carcinoma, plasmacytoma of the occipitocervical joint, and a sarcoid lesion. We classified tumors into extracranial, intradural, intraosseous, and dumbbell-shaped, and analyzed the approach selection for each.

Conclusion

Jugular foramen and posterolateral skull base lesions can be safely resected through a retro-auricular distal cervical lateral skull base approach, which is customizable to anatomical location and tumor extension by tailoring the involved osteo-muscular triangles.

## Introduction

Surgical access to distal cervical and jugular foramen (JF) lesions is difficult given the anatomical complexity of the region, technical challenges, and risk of neurovascular injuries. Approach selection is key in devising the surgical strategy and achieving complete resection. Given the usual depth of the pathology, muscular and bony structures should be unlocked in a stepwise and targeted fashion. Tailoring the approach to the individual case allows for adequate exposure around the lesion while minimizing unnecessary steps, wasted time, and risk of collateral injuries.

Tumor extension has been classified based on pathological diagnosis and its anatomical extension (glomus tumors, cranial nerve schwannomas, meningiomas) [1-7]. Several classification schemes have also been proposed to help plan the surgical approach. Dr. Albert Rhoton Jr. classified the JF approaches into an anterior group through the tympanic bone (preauricular subtemporal-infratemporal fossa approach), a lateral group through the mastoid bone (post-auricular transtemporal infralabyrinthine approach, and other transmastoid approaches), and a posterior group (retrosigmoid, far lateral and trans-condylar approaches) [8]. The senior author (JHR) traditionally adopted the lateral distal cervical retro-auricular transtemporal (LDC-RATT) approach because it offers direct access to the JF, is versatile, and can be tailored to individual lesions [9,10]. We recently deconstructed this approach into anatomical triangles and analyzed its different angles of exposure [11].

In this paper, we review a clinical series of 32 lesions where a modification of the LDC-RATT was used by the senior author (JHR) for surgical resection of distal cervical and posterolateral skull base lesions, either extending to or in close proximity to the jugular foramen. We classified these according to their anatomical extension and analyzed the anatomical factors influencing approach selection and modification. Such a classification was combined with an illustration of the jugular foramen triangles in planning and executing the approach.

## Materials and methods

Anatomical dissections

Six preserved cadaveric heads (12 sides) were dissected at the Medical Education and Research Institute (MERI) microsurgical laboratory in Memphis, TN. The heads had no known intracranial or cervical pathology and were fixed and injected with colored silicone rubber. All cadaver specimens had preserved distal necks proximally, at least to the level of the omohyoid muscle.

**Figure 1:**
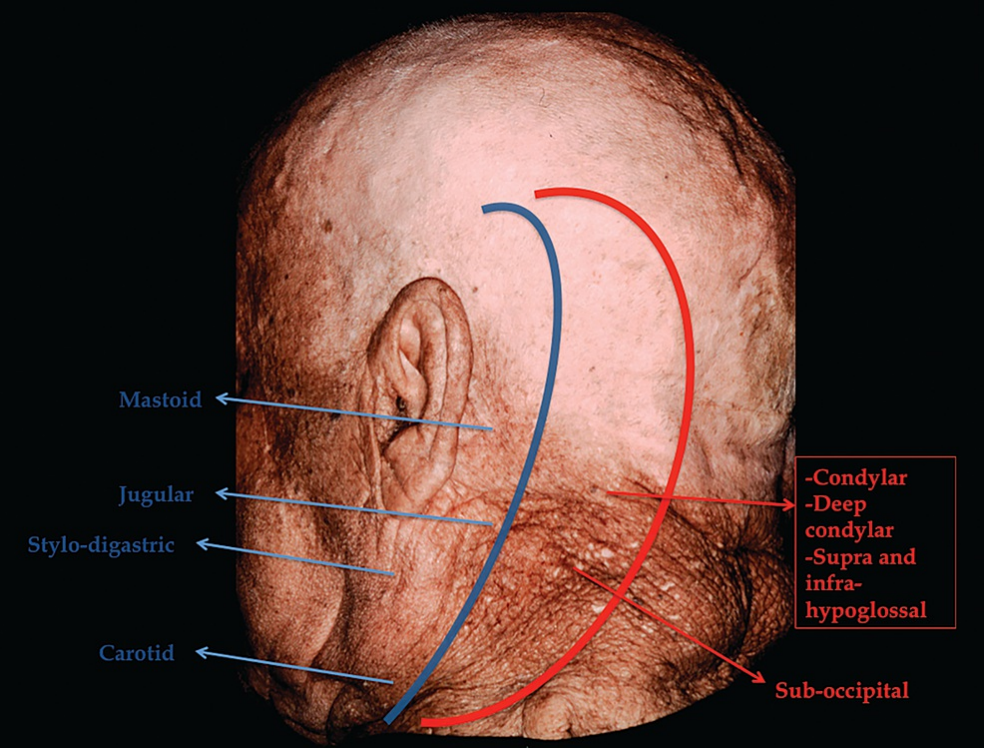
Planning the lateral distal cervical retro-auricular trans-temporal approach in relation to anatomical triangles. To expose the jugular foramen (blue), the approach unlocks the carotid, stylo-digastric, jugular, and mastoid triangles. A modified postero-lateral approach (red) extends the exposure medially to the condylar region, leading to the condylar, deep condylar, suboccipital, supra-hypoglossal, and infra-hypoglossal triangles.

**Figure 2:**
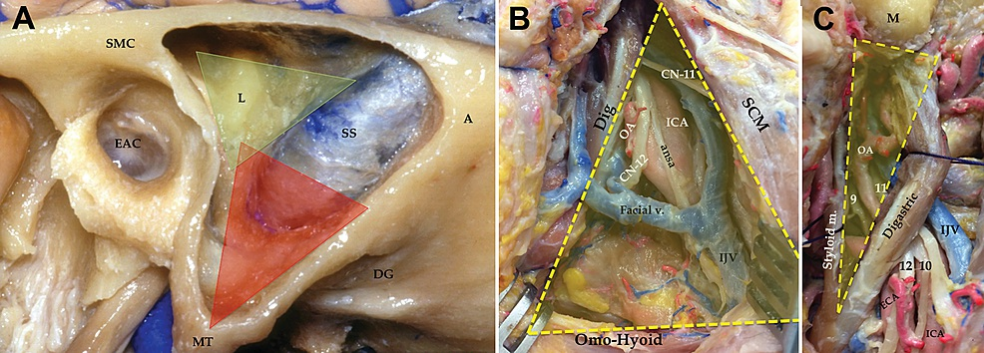
Mastoid, carotid, and stylodigastric triangles. (A) *Mastoid triangle: *drilled between the supra-mastoid crest (SMC), asterion (label A), and mastoid tip (MT). It can be divided into an infra-labyrinthine or supra-jugular sector (red), and a pre-sigmoid sector (yellow). Labels: A: asterion; SMC: supra-mastoid crest; DG: digastric groove; EAC: external auditory canal; L: labyrinth; MT: mastoid tip; SS: sigmoid sinus. (B) *Carotid triangle: *limited by the sternocleidomastoid (SCM), digastric (Dig), and omohyoid muscles. Labels: ansa: ansa cervicalis; CN-11: accessory nerve; CN-12: hypoglossal nerve; Dig: digastric muscle; ICA: internal carotid artery; IJV: internal jugular vein; OA: occipital artery; SCM: sternocleidomastoid. (C) *Stylodigastric triangle: *defined by the (a) digastric muscle, (b) styloid muscles, and an imaginary line between the digastric groove and base of the styloid process. Labels: 9=glossopharyngeal nerve; 10=vagus nerve; 11=accessory nerve; 12=hypoglossal nerve; ECA: external carotid artery; IJV: internal jugular vein; M: mastoid process.

**Figure 3:**
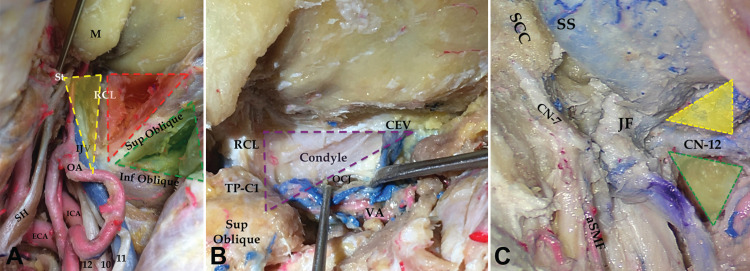
Suboccipital, condylar, jugular, supra-hypoglossal, and infra-hypoglossal triangles. (A) *Suboccipital triangle *(green): limited by the superior and inferior obliques muscles. *Condylar triangle *(red): between the superior oblique and the rectus capitis lateralis (RCL) muscles. *Jugular triangle *(yellow): limited by the RCL, transverse process of the atlas, and the base of the styloid (St). (B) *Deep condylar *and *trans-condylar triangles:* defined between the condylar emissary vein (CEV), RCL, and transverse process of the atlas (TP-C1). The atlanto-occipital joint (or occipitocervical joint, OCJ) is seen after mobilizing and retracting the vertebral artery (VA) inferiorly. (C)* Supra-hypoglossal *(yellow) and *infra-hypoglossal *(green) *triangles: *delineated after drilling the occipital condyle with the former leading to the infrajugular area, and the latter to the occipitocervical joint. Labels: aSMF: artery of stylomastoid foramen; CN-7: facial nerve; CN-12: hypoglossal nerve; JF: jugular foramen.

Two types of retro-auricular curvilinear C-shaped skin incisions were made posterior to the pinna: either 3 cm posterior to the pinna for a standard lateral approach, or further medially close to the midline in order to expose the occipital condyle (modified approach with a far lateral component; Figure 1). Three major landmarks were constantly recognized: the mastoid tip, styloid process, and transverse process of the atlas (TP-C1). The following superficial triangles were defined and identified as previously described (Figures 2 and 3): (i) *mastoid triangle*, between the asterion, mastoid tip, and the supra-meatal crest (Figure 2A) [12]; (ii) *carotid triangle*, limited by the omohyoid, the sternocleidomastoid (SCM), and the digastric muscles (Figure 2B) [13]; and (iii) *stylodigastric triangle *between the digastric and the stylo-hyoid muscles (Figure 2C) [12].

Detaching the digastric muscle and mobilizing it inferiorly exposed the TP-C1 and the surrounding deep muscular triangles: (iv) *suboccipital triangle *between the superior and inferior oblique muscles (Figure 3A) [14], and (v) *condylar triangle *between the superior oblique and rectus capitis lateralis muscles (Figure 3A) [15]. We also previously defined the (vi)* “jugular triangle” *between rectus capitis lateralis, TP-C1, and the base of the styloid process, as the lateral limit to the jugular foramen (Figure 3A) [11]. We proposed the (vii) *“deep condylar triangle” *between the condylar emissary vein, the jugular process of the occipital bone (site of insertion of rectus capitis lateralis), and the transverse process of the atlas (Figure 3B) [11]. This helped us delineate exactly where the occipital condyle is located from a lateral perspective. Drilling the occipital condyle led us to the hypoglossal canal. Above it, the (viii) *supra-hypoglossal triangle *was defined between the hypoglossal dura inferiorly, foramen magnum dura medially, and jugular bulb laterally. Below it, the (9) *infra-hypoglossal triangle *was identified between the hypoglossal dura, atlanto-occipital joint, and jugular foramen (Figure 3C) [16].

Case series

We retrospectively reviewed illustrative cases in which different modifications of the LDC-RATT approach were used by the senior author (JHR) at our institution between 2000 and 2016. Emphasis was placed on challenging lesions, such as glomus jugulare tumors, lower cranial nerve schwannomas, jugular foramen meningiomas, and occipital condyle and/or atlanto-occipital joint lesions extending to the jugular foramen.

Pre-operative imaging (including computed tomography [CT] scans, magnetic resonance imaging [MRI], and, if available, four-vessel angiography) and intraoperative findings were reviewed on 31 patients treated surgically for 32 such lesions. We focused on the anatomical factors of these lesions, which were deemed crucial in planning the surgical approach. We obtained Institutional Review Board approval from The University of Tennessee Health Science Center (UTHSC) (No. 20-07874-XP). Informed consent was waived because of the retrospective nature of the data.

**Figure 4:**
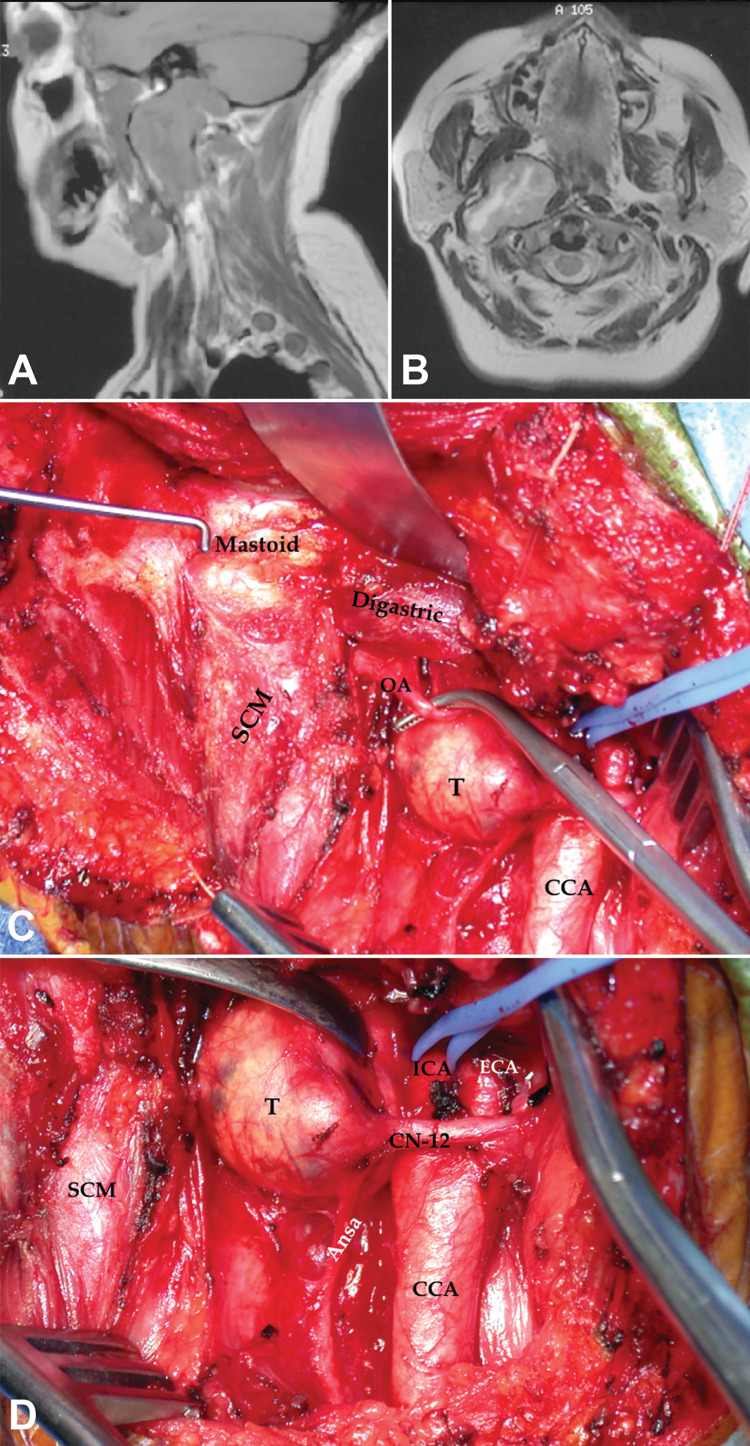
Dumbbell-shaped hypoglossal schwannoma accessed via carotid triangle. T1 sagittal (A) and T2 axial (B) MRI scan of a right-sided triple dumbbell-shaped hypoglossal schwannoma in a patient in their early 40s who presented with headaches, nausea, vomiting, and right tongue atrophy (case 31). The patient underwent a combined approach with antero-lateral neck dissection, exposing a tumor in the carotid triangle (C, D), followed by a retrosigmoid craniotomy. Labels: ansa: ansa cervicalis; CCA: common carotid artery; CN-12: hypoglossal nerve; ECA: external carotid artery; ICA: internal carotid artery; OA: occipital artery; T: tumor.

**Figure 5:**
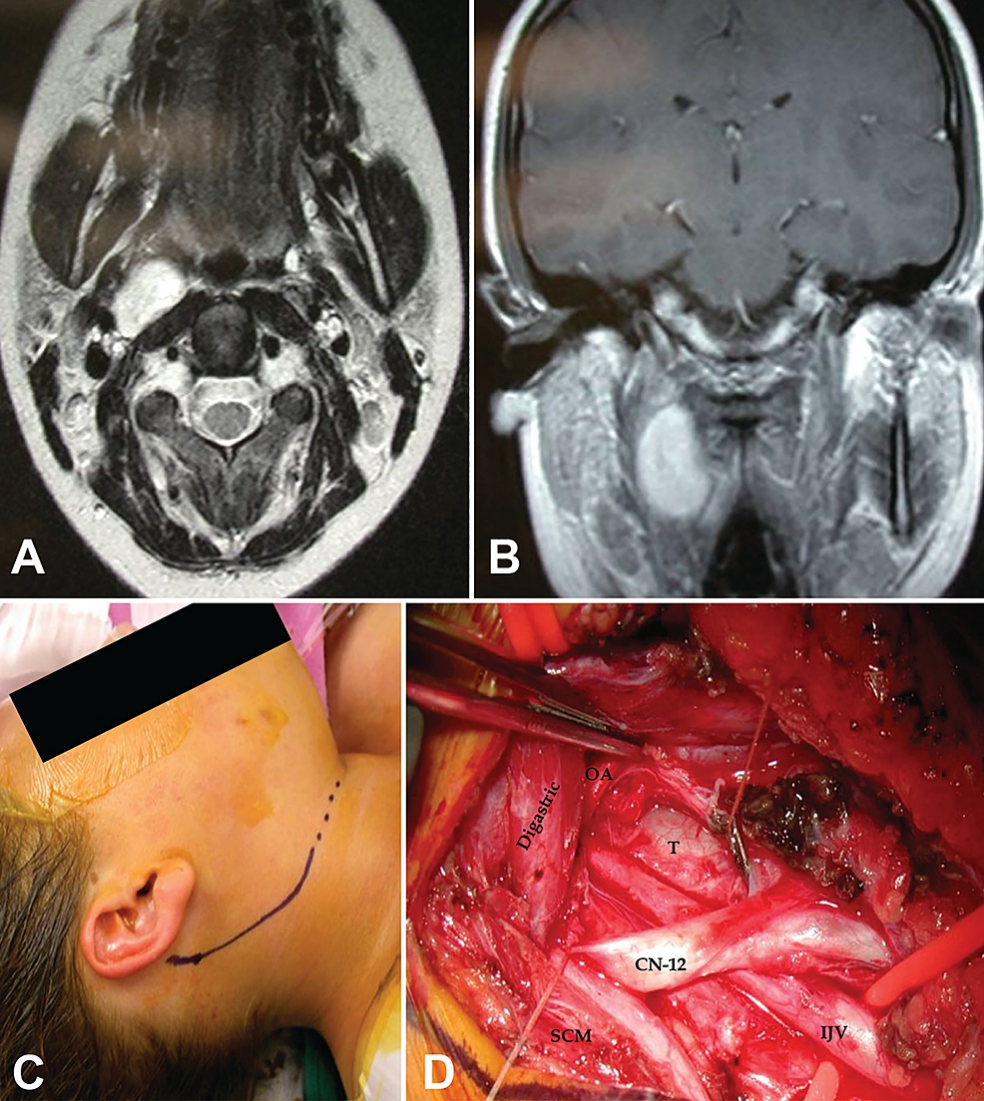
Glossopharyngeal schwannoma accessed via stylodigastric triangle. Axial T2 (A) and coronal T1 (B) gadolinium-enhanced MRI of a glossopharyngeal schwannoma in a pediatric patient with neurofibromatosis type-1 presenting with a neck mass (case 4). Note the tumor’s location is distal in the neck and medial to the internal carotid artery. A distal lateral cervical exposure was performed (C, D) and the stylodigastric triangle opened by mobilizing the digastric muscle. Labels: CN-12: hypoglossal nerve; IJV: internal jugular vein; OA: occipital artery; SCM: sternocleidomastoid muscle; T: tumor.

**Figure 6:**
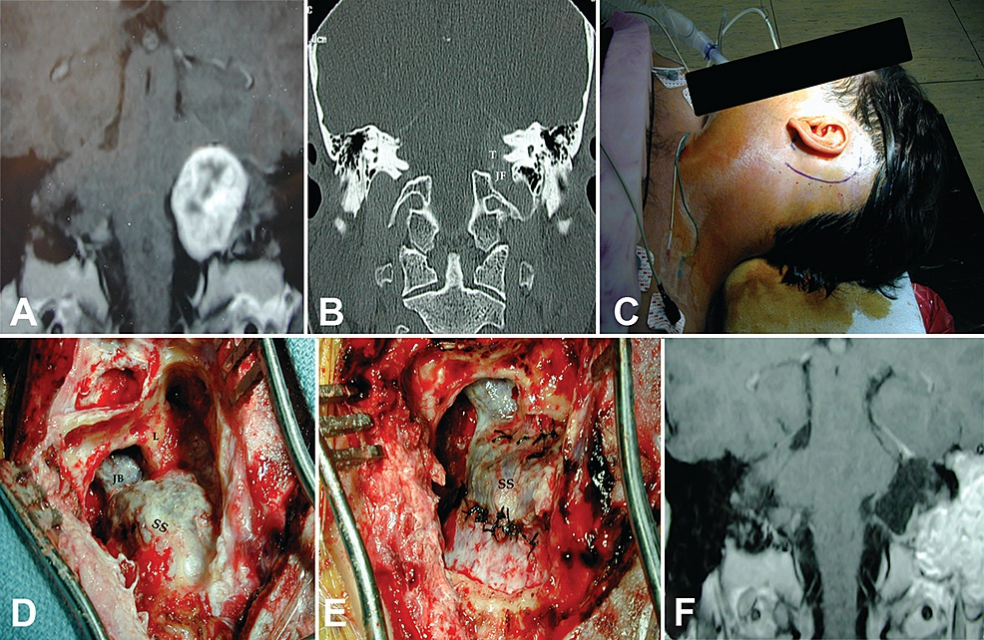
Glossopharyngeal schwannoma accessed via mastoid triangle. Coronal T1 gadolinium-enhanced (A) and computed tomographic scan (B) of a mostly intradural glossopharyngeal schwannoma (case 8). The patient in their late 30s presented with hearing loss, tinnitus, and vertigo, and the tumor was mostly intracranial impinging toward the jugular foramen (type 2). A retroauricular incision was planned (C) and the mastoid triangle was exposed in both its presigmoid and suprajugular sectors (D). A combined presigmoid and retrosigmoid intradural approach was performed (E) to achieve a gross total resection, as seen on the postoperative coronal MRI scan (F). Labels: JB: jugular bulb; L: labyrinth; SS: sigmoid sinus.

**Figure 7:**
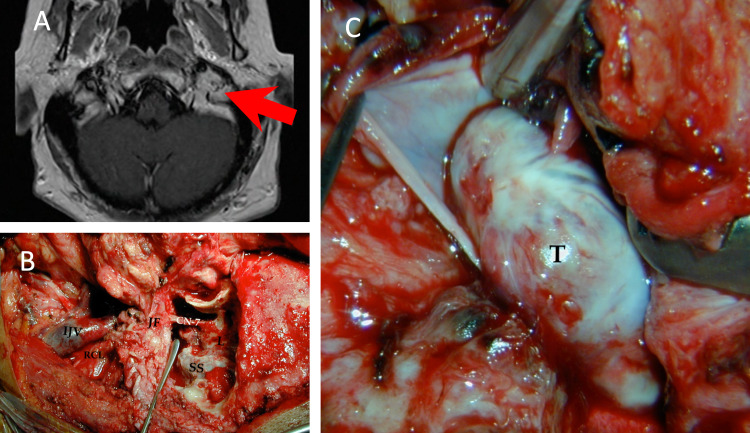
Glomus jugulare tumor accessed via jugular triangle. Exposure of the deeper surgical triangles, including the jugular triangle. (A) Axial T1 MRI scan demonstrating a left glomus jugulare tumor in a patient in their mid-40s presenting with hearing loss and pulsatile tinnitus (case 9). A lateral distal cervical retro-auricular transtemporal (infra-labyrinthine) approach was performed to expose the jugular foramen (JF) (B). The fallopian canal was skeletonized keeping a bone shell around the facial nerve (CN-7) to preserve it. After ligating the sigmoid sinus and the jugular vein, the vein was opened, and the tumor resected from within (C). The medial wall of the vein was preserved to protect the neighboring lower cranial nerves. Labels: CN-7: facial nerve; IJV: internal jugular vein; JF: jugular foramen; L: labyrinth; RCL: rectus capitis lateralis; SS: sigmoid sinus.

**Figure 8:**
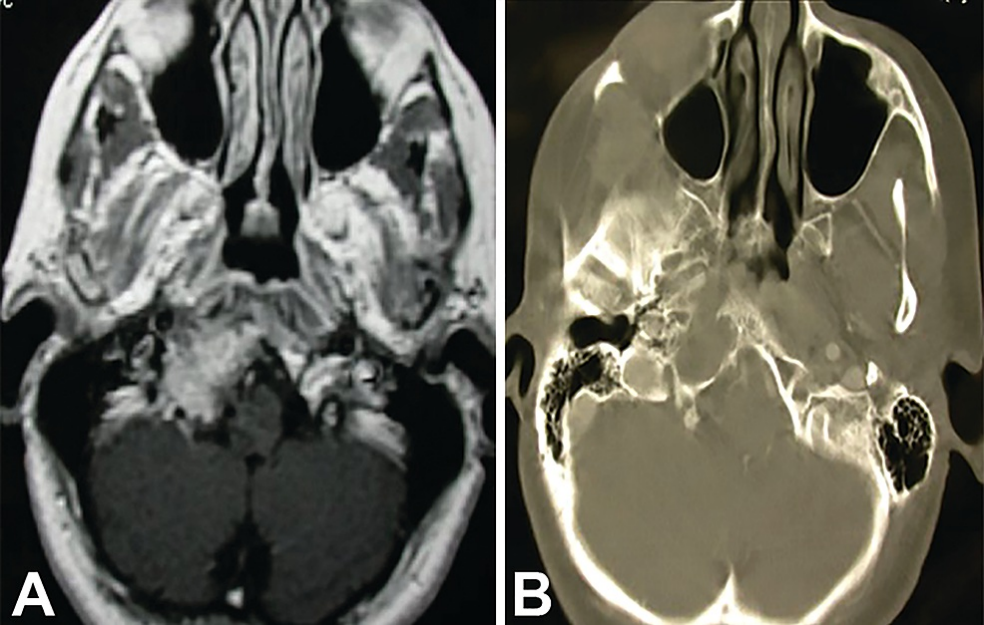
Plasmacytoma accessed via infra-hypoglossal triangle. (A) A contrasted T1 axial MRI and (B) axial CT scan demonstrating a lesion in the right occipitocervical joint. The patient complained of severe right-sided neck pain, and a modified approach was done exposing the lesion in the infra-hypoglossal triangle. Pathology was consistent with plasmacytoma (case 16).

**Figure 9:**
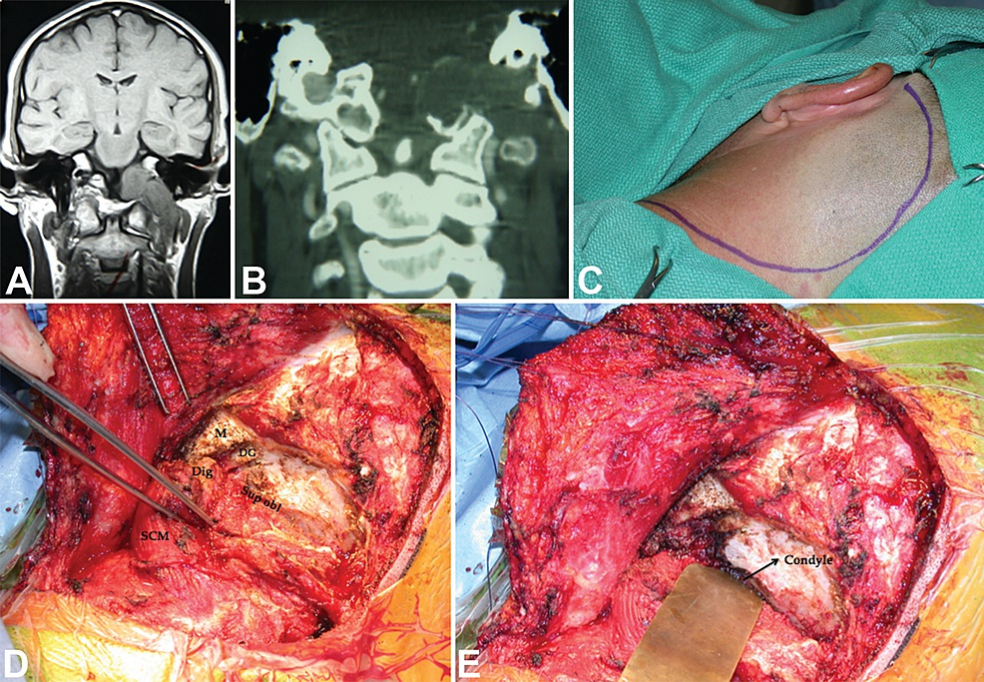
Hypoglossal schwannoma accessed via condylar triangle. Coronal T1 MRI (A) and coronal CT (B) scans showing a left hypoglossal schwannoma involving the cerbello-medullary cistern, occipital condyle, and hypoglossal canal, medial jugular foramen, and the distal cervical region. The 37-year-old man presented with headache, left neck pain, and hemiatrophy of the left tongue (case 32). A modified approach that combined a far lateral exposure was done (C, D, E) to identify the condylar triangle under the superior oblique muscle (sup obl). The intracranial, intraforaminal, and cervical components of the tumor were then completely resected. Labels: DG: digastric groove; Dig: digastric muscle; M: mastoid; SCM: sternocleidomastoid, Sup obl: superior oblique muscle.

Anatomically, lesions were classified according to their origin and extension (Table 1). Extracranial tumors (type 1) are limited to the distal cervical region. While type 1A lesions are located below the digastric muscle in the carotid triangle (Figures 2B and 4), type 1B tumors are in the stylodigastric triangle close to the skull base (Figures 2C and 5). Type 2 tumors are intracranial and involve at least the cerebello-medullary and/or the cerebello-pontine cisterns (Figure 6). Type 3 tumors are typically located at the level of the skull base and are termed intra-osseous or intraforaminal. Type 3A is limited to the jugular foramen (Figure 7), while type 3B lesions extend to the petrous bone and/or the internal auditory meatus, and type 3C tumors to the occipital condyle region (Figure 8A-8B). Type 4 tumors are dumbbell-shaped, with 4A involving the jugular foramen and 4B the medial aspect of the jugular foramen and hypoglossal canal (Figure 9A-9B). Type 4 tumors can be true or triple dumbbell-shaped (4t) involving the extracranial, foraminal, and intracranial compartments (Figures 5A-5B and 9A-9B); predominantly foraminal and extra-cranial (4e); or foraminal and intracranial (4i).

**Table 1:** Classification of posterolateral skull base/jugular foramen lesions depending on their anatomical extension. IAC: internal auditory meatus; OC: occipital condyle.

Group 1: Extracranial
1A Carotid triangle
1B Stylodigastric triangle
Group 2: Intracranial Intradural
Group 3: Intraosseous or Intraforaminal
3A Jugular foramen
3B Petrous/IAC
3C Hypoglossal canal/OC
Group 4: Dumbbell-shaped
4A Jugular foramen
4Ae Predominant extracranial
4Ai Predominant intracranial
4At True dumbbell-shaped
4B Hypoglossal canal/OC
4Be Predominant extracranial
4Bi Predominant intracranial
4Bt True dumbbell-shaped

We then analyzed the different modifications of the LDC-RATT approach as employed in clinical practice by the senior author (JHR), compared to our anatomical dissections and of the described surgical triangles.

## Results

Anatomical dissections

Contents of the different triangles along with their respective measurements have been previously reported [11]. Table 2 summarizes the triangles as they relate to the modifications of the LDC-RATT approach, including the different possible surgical steps and exposure advantages related to the jugular foramen region.

**Table 2:** Surgical triangles deconstructing the lateral distal cervical retro-auricular transtemporal approach, and the potential steps involved in each. EAC: external auditory meatus; HC: hypoglossal canal; IAC: internal auditory meatus; ICA: internal carotid artery; IJV: internal jugular vein; JB: jugular bulb; JF: jugular foramen; LCN: lower cranial nerves (CN9–12); OC: occipital condyle; RCL: rectus capitis lateralis; SCM: sternocleidomastoid muscle; VA: vertebral artery.

Triangle	Major surgical steps	Exposure to JF region
1. Carotid	Medial SCM approach and carotid sheath dissection	Proximal control, distal course of LCN’S
2. Stylodigastric	Mobilizing/detaching digastric muscle	Lateral JF and distal cervical region, distal ICA
	Removing styloid process	Anterior JF, distal ICA
3. Mastoid	Infralabyrinthine	Lateral and superior JF
	Retrolabyrinthine, translabyrinthine, transcochlear	Superior JF and JB, IAC and petrous bone, intradural exposure
4. Jugular	RCL and jugular process	Posterior JF
	Cutting the jugular ring	Medial JF
5. Condylar	Detaching superior oblique muscle	Expose the OC
6. Suboccipital	Detach the superior and inferior oblique	Expose VA
7. Deep condylar	Drill the OC	Medial JF, HC
8. Supra-hypoglossal	Drill the jugular tubercle	Inferior JF Premedullary cistern
9. Infrahypoglossal	Drill below the hypoglossal canal	Expose the atlanto-occipital joint

A *standard LDC-RATT* combines a distal medial sternocleidomastoid approach with a retro-auricular transmastoid approach. The incision is usually started behind the pinna and continued inferiorly over a neck crease on the medial aspect of the SCM. The distal cervical component of the approach is divided into a carotid triangle (harboring the proximal cervical internal carotid artery [ICA], external carotid artery [ECA], and common carotid artery [CCA]; internal jugular vein [IJV]; ansa cervicalis, and lower cranial nerves [CN] X-XII) and a stylodigastric triangle (containing the distal ICA, distal IVJ, distal ECA, and its branches, and CN IX-XII), separated by the digastric muscle. Extracranial schwannomas or distal IJV/ICA lesions can be accessed through these triangles (Figures 2B, 2C, 4, and 5).

Both the digastric and SCM muscles attach to the mastoid bone and should be reflected if further steps of the approach are necessary. Drilling the mastoid bone can be compartmentalized into an infralabyrinthine space (or “suprajugular space” to expose the superior aspect of the jugular foramen and jugular bulb), and a retrolabyrinthine/translabyrinthine space (leading to the presigmoid dura; Figures 2A and 6). The IJV enters the JF in the jugular triangle (TPC1-rectus capitis lateralis-base of the styloid process). Thus, exposing a standard glomus jugulare tumor requires opening the carotid, stylodigastric, mastoid, and jugular triangles (Figures 3A and 7). Palpating the transverse process of the atlas, the styloid process, and the mastoid tip is helpful in orienting the surgeon toward the correct triangles.

A *modified LDC-RATT* is helpful for lesions involving the region of the occipital condyle and medial aspect of the jugular foramen. This approach adds a far lateral exposure and is devised by curving the retro-auricular incision further medially. It allows the exposure of the occipital bone beyond the occipitomastoid suture and the suboccipital triangle beneath it. Again, the transverse process of the atlas represents a key anatomical structure that helps delineate the suboccipital triangle (where the vertebral artery is identified), and the condylar triangle (harboring the occipital condyle [OC]). The OC is delineated exactly in the deep condylar triangle, and drilling it exposes the hypoglossal canal. The supra-hypoglossal triangle aids in exposing the inferior aspect of the jugular foramen (described in the literature as the infrajugular approach), while the infra-hypoglossal triangle leads to the atlanto-occipital joint (Figures 3B, 3C, and 8).

Case series

The confirmed pathological diagnosis of the reviewed lesions included: 13 lower cranial nerve schwannomas or neurofibromas (eight hypoglossal, two vagal, two glossopharyngeal, and one accessory), seven glomus jugulare/vagale tumors, seven meningiomas (mostly petrous or petroclival extending into the jugular foramen), one chondrosarcoma, one adenocystic carcinoma, one plasmacytoma of the occipitocervical joint, one eagle syndrome (hypertrophied styloid process compressing the internal carotid artery and causing transient ischemic attack [TIA] like syndrome), and one sarcoid lesion in the occipital condyle/medial jugular foramen area. Table 3 summarizes the clinical presentation, pathology, employed surgical approach, and extent of resection in each of these cases.

**Table 3:** List of lesions treated with a modification of the distal cervical retro-auricular jugular foramen approach by the senior author (JHR). CMC: cerebello-medullary cistern; CN: cranial nerve; CPA: cerebello-pontine angle; GKS: gamma knife surgery; GTR: gross total resection; HC: hypoglossal canal; IAC: internal auditory canal; JF: jugular foramen; LDC-RATT: lateral distal cervical retroauricular transtemporal approach; NF: neurofibromatosis; NTR: near-total resection (90–99% resection); NV: nausea/vomiting; SD: stylo-digastric; STR: subtotal resection (<90%); translab: translabyrinthine.

Case	Presentation	Pathology	Location	Type	Approach	Triangles	EOR
1	Neck mass/pain	CN12 schwannoma	Lateral cervical	1A	Lateral cervical	Carotid	GTR
2	Tongue atrophy	CN12 schwannoma	Lateral cervical	1A	Lateral cervical	Carotid	GTR
3	Hoarseness, tongue atrophy	CN10 schwannoma	Distal cervical	1B	Distal cervical	Carotid, SD	GTR
4	NF1, neck mass	CN9 schwannoma	Distal cervical	1B	Distal cervical	Carotid, SD	GTR
5	NF1, neck mass	CN10 neurofibroma	Distal cervical	1B	Distal cervical	Carotid, SD	GTR
6	Trapezius weakness/pain	CN 11 schwannoma	Distal cervical	1B	Distal cervical	Carotid, SD	GTR
7	Transient ischemic attacks	Hypertrophic styloid process (Eagle syndrome)	Distal cervical	1B	Distal cervical	Carotid, SD	GTR
8	Hearing loss, tinnitus, vertigo	CN9 schwannoma	CPA and CMC	2	Combined presigmoid and retrosigmoid	Mastoid	GTR
9	Hearing loss, tinnitus	Glomus jugulare	JF	3A	LDC-RATT	Carotid, SD, mastoid, jugular	GTR
10	Headaches, tinnitus	Glomus jugulare	JF	3A	LDC-RATT	Carotid, SD, mastoid, jugular	GTR
11	Headaches, tinnitus	Glomus jugulare	JF	3A	LDC-RATT	Carotid, SD, mastoid, jugular	GTR
12	Severe headaches/ neck pain	Sarcoid	JF	3A	LDC-RATT	Carotid, SD, mastoid, jugular	GTR
13	Hearing loss, headaches, tinnitus	Glomus jugulare	JF + petrous bone	3B	LDC-RATT/translab	Carotid, SD, mastoid, jugular	NTR
14	Hearing loss, facial weakness, dizziness	Glomus jugulare	JF + petrous bone	3B	LDC-RATT/translab	Carotid, SD, mastoid, jugular	NTR
15	headaches, hearing loss	Chondrosarcoma	JF + petroclival	3B	LDC-RATT/translab	Carotid, SD, mastoid, jugular	NTR
16	Severe neck pain	plasmacytoma	Condyle, medial JF	3C	Modified LDC-RATT + far lateral	Condylar, suboccipital	GTR
17	Pulsating neck mass, CN9–12 palsies	Glomus vagale	JF+distal cervical	4Ae	Distal cervical + GKS	Carotid, SD	STR
18	CN10–11 palsies	CN10 schwannoma	JF+CPA	4Ai	LDC-RATT/translab/presigmoid	Carotid, SD, mastoid, jugular	NTR
19	Ataxia, CN8–12 palsies, hearing loss	Glomus with aggressive behavior	JF+CPA	4Ai	LDC-RATT/transcochlear/presigmoid	Carotid, SD, mastoid, jugular	STR
20	Headaches, ataxia	Petrous/JF meningioma	CPA, CMC, JF	4Ai	Retrosigmoid	Mastoid, jugular	NTR
21	Headaches, ataxia, hearing loss	Petrous/JF meningioma	CPA, CMC, IAC, JF	4Ai	Retrosigmoid	Mastoid, jugular	STR
22	Headaches, ataxia	Petrous/JF meningioma	CPA, CMC, JF	4Ai	Retrosigmoid	Mastoid, jugular	NTR
23	Headaches, ataxia, tinnitus	Petrous/JF meningioma	CPA, CMC, JF	4Ai	Retrosigmoid	Mastoid, jugular	NTR
24	Hearing loss, ataxia, facial numbness	Petroclival/JF meningioma	CPA, CMC, IAC, JF	4Ai	Retrosigmoid followed by GKS	Mastoid, jugular	STR
25	Hearing loss, ataxia, facial numbness	Petroclival/JF meningioma	CPA, CMC, IAC, JF	4Ai	Extended petrosal	Mastoid, jugular	GTR
26	Neck mass, CN9–11 palsies	Petrous/ JF meningioma	CPA/IAC, CMC, JF, distal cervical	4At	Stage1: distal cervical; Stage 2: retrosigmoid	Carotid, SD, mastoid, jugular	STR
27	Ataxia, headaches, neck mass, hearing loss	Adenoid cystic carcinoma	CPA, CMC, JF, distal cervical	4At	LDC-RATT/translab	Carotid, SD, mastoid, jugular	NTR
28	Headaches, CN12 palsy	Hypoglossal schwannoma	HC/condyle, medial JF, distal cervical	4Be	Modified LDC-RATT + far lateral	SD, condylar, jugular	GTR
29	Headaches, CN12 palsy	Hypoglossal schwannoma	HC/condyle, medial JF, distal cervical	4Be	Modified LDC-RATT + far lateral	SD, condylar, jugular	GTR
30	Headaches, CN12 palsy	Hypoglossal schwannoma	CMC, HC/condyle, medial JF	4Bi	Modified LDC-RATT + far lateral	Condylar, suboccipital, jugular	NTR
31	Headaches, NV, CN12 palsy	Hypoglossal schwannoma	CMC, JF, HC, distal cervical	4Bt	Modified LDC-RATT + retrosigmoid	Carotid, SD, jugular, mastoid, condylar	STR
32	Headaches, neck pain, CN12 palsy	Hypoglossal schwannoma	CMC, HC/condyle, medial JF, distal cervical	4Bt	Modified LDC-RATT + far lateral	Carotid, SD, jugular, condylar	GTR

Type 1A tumors were approached through the carotid triangle. For more distal lesions beyond the angle of the jaw or the digastric muscle (type 1B), the stylodigastric triangle was opened by either mobilizing or detaching the digastric muscle (Figures 2B, 2C, 4, and 8). Intradural tumors (type 2) were mostly located intracranially with limited intraforaminal extension. These were approached through the presigmoid, retrosigmoid, or lateral suboccipital (or far lateral) corridors (Figures 2A and 6). Intraosseous tumors (type 3) were located in the skull base (jugular foramen, occipital condyle, lower part of the mastoid, and petrous bone). These were accessed through the jugular triangle using a classic LDC-RATT approach if the origin of the tumor was believed to be in the jugular foramen (type 3A; Figures 2d and 7). Extension along the petrous bone or to the petroclival area (type 3B) dictated a petrosal component to the approach (retrolabyrinthine, translabyrinthine, or a transcochlear approach). A modified LDC-RATT or a far lateral approach with a medially oriented incision, leading to the condylar triangles, was performed whenever the occipital condyle and medial aspect of the jugular foramen were compromised (type 3C; Figures 3c, 8a, and 8b).

Dumbbell-shaped tumors (type 4) required either combined or staged procedures through variations of the retro-auricular approach (Figures 2b and 4). A translabyrinthine, transcochlear, or retrosigmoid part was added to the LDC-RATT approach for type 4Ai tumors to further address the intracranial disease. Stereotactic radiosurgery was helpful to control the foraminal component after either removing the symptomatic intracranial (4Ai) or distal cervical parts (4Ae) in other cases.

True jugular dumbbell-shaped tumors (type 4At, comprising all intracranial, foraminal, and extracranial compartments) were treated with a staged distal cervical followed by retrosigmoid resection, or a combined LDC-RATT and translabyrinthine approach. Type 4B tumors (extending to the condylar triangle) were treated with a modified LDC-RATT approach combined with either a suboccipital retrosigmoid or an intradural far lateral approach (Figures 4 and 8).

In summary, important anatomical factors found to be highly influential on selecting the surgical approach included the following: displacement of the distal cervical internal carotid artery (if the tumor moved the artery anteriorly, this dictated a posterolateral, rather than anterior, approach); involvement of the occipital condyle; tumor extension to the petrous bone or to the internal auditory meatus; tumor vascularity and origin of arterial feeders; patency of the sigmoid sinus; and preoperative cranial nerve deficits (described in the Discussion section).

## Discussion

Fisch classified glomus tumors according to their anatomical extension: middle ear (type A), tympanomastoid complex (B), labyrinthine and petrous areas (C), and intracranial (D) [1,2]. Glasscock-Jackson adopted a similar organization [9]. Fukushima classified jugular foramen schwannomas into intracranial, dumbbell-shaped, and extracranial [3], while he divided hypoglossal schwannomas into intradural, dumbbell-shaped, skull base extracranial, and peripheral tumors (completely extra-cranial) [7]. Similarly, Samii et al. divided jugular foramen schwannomas into intracranial or cisternal tumors (type A); intraosseous tumors (B); extracranial tumors (type C); and triple dumbbell-shaped tumors, depending on the employed surgical approach [4].

Given the overlap in surgical approaches and anatomical extensions, we combined previous classifications to help systematize posterolateral skull base and distal cervical lesions, regardless of their pathological diagnosis, in one scheme. Most of these lesions can be simplified into extracranial, intracranial, intraosseous/intraforaminal (at the level of the skull base), and dumbbell-shaped. We believe that this is helpful to tailor the surgical approach and its steps to each lesion and avoid the risks of radical exposures if not necessary.

**Figure 10:**
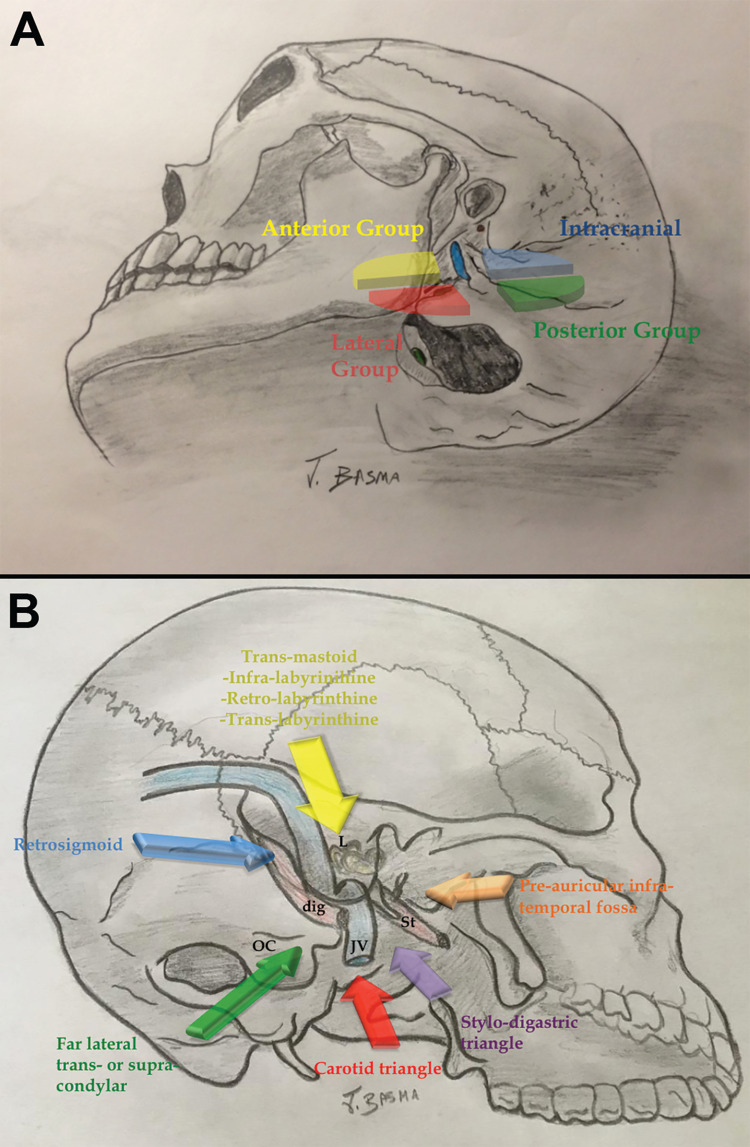
Jugular foramen approaches. (A) Illustration depicting an adapted classification of jugular foramen approaches by Dr. Albert Rhoton Jr. Intracranial approaches can be performed either through the posterior group (retrosigmoid or far lateral), or lateral group (presigmoid transmastoid approach). (B) Another illustration elucidates the different angles of attack to the jugular foramen as they relate to the lateral distal cervical retro-auricular trans-temporal (LDC-RATT) approach. The lateral cervical dissection includes exposure through the carotid (red) and/or stylodigastric triangles (purple) with the latter overlapping part of—and in many instances supplanting—the anterior pre-auricular infratemporal fossa approach (orange). The transmastoid component of the approach (yellow) provides access to the superior access of the jugular foramen (infralabyrinthine approach). Intracranial exposure can be obtained by combining a presigmoid corridor (retro- and trans-labyrinthine approach) or a retrosigmoid craniotomy (blue). To extend the approach posteromedially, the rectus capitis lateralis can be mobilized and the jugular process drilled (para-condylar exposure). A more medial incision can incorporate a far lateral and trans-condylar (green) component if necessary. Labels: dig: digastric, JV: jugular vein, L: labyrinth, OC: occipital condyle, St: styloid process.

On the other hand, Fisch divided the post-auricular approach itself into types A, B, and C based on the required anterior exposure. In the classic type A approach, a distal cervical dissection is combined with a radical mastoidectomy, with further transposition of the facial nerve to expose the posterior aspect of the infratemporal fossa and petrous carotid. While the type B approach extends to the petrous apex and clivus, type C widens the exposure to include the anterior aspect of the middle fossa and infratemporal fossa [17]. The post-auricular approach described by Fisch falls in the lateral group of approaches as classified by Rhoton (post-auricular transtemporal infralabyrinthine approach, and other transmastoid approaches) (Figure 10A) [8]. However, it is more extensive and includes radical steps associated with a higher rate of complications, such as facial nerve transposition.

The lateral approach (i.e., LDC-RATT) is versatile and can be customized to the specific anatomical location of the lesion (Figure 10B) [11]. The styloid process may be removed to gain part of the exposure obtained from a preauricular subtemporal-infratemporal fossa approach (anterior group) [8,18]. The rectus capitis lateralis can be detached and the jugular process of the occipital bone drilled, overlapping with the precondylar variation of the far lateral approach (posterior group) [19]. In a modified LDC-RATT approach, the exposure is combined with a far lateral approach to access the condylar area [20].

To this end, we deconstructed the approach into sequential steps in predictable anatomical triangles (Table 2 and Figure 1). In a similar way with cavernous sinus and middle fossa triangles, these can help compartmentalize the lesion and the operative stages required to reach it. Given the preponderance of radiosurgery and the rarity of these lesions, younger neurosurgeons are becoming less familiar with the subtle nuances of approaching jugular foramen pathologies. Lesions are sometimes deemed inoperable or inaccessible given a poor approach selection. Although not necessary, the triangles can provide young neurosurgeons who are not familiar with the anatomy, with a diagrammatic tool until more intuitive knowledge is developed.

Besides the anatomical relationship with described surgical triangles, several anatomical factors were found to be crucial in deciding the surgical approach. If the internal carotid artery is displaced posteriorly, then an anterior jugular foramen approach needs to be considered, such as opening the stylodigastric triangle and resecting the styloid process (Figures 2C and 5). However, if the tumor is further anterior in the infratemporal fossa, then a pre-auricular infratemporal fossa approach should be undertaken [18].

Involvement of the occipital condyle and the hypoglossal canal is key in deciding on the medial extent of the approach (Figures 3B, 3C, and 9). In such instances, and even if the tumor originates in the jugular foramen, exposure is carried medially to the transverse process of the atlas in order to allow visualization of the superficial and deep condylar triangles. If the tumor originates more medially toward the foramen magnum, then a more medial approach may be selected (suboccipital or far lateral approach) [14].

Mastoid bone involvement can be divided into two major sectors: infralabyrinthine (or suprajugular) space and a presigmoid sector (retrolabyrinthine, translabyrinthine, and transcochlear approaches) [16]. An infralabyrinthine mastoidectomy usually allows good exposure of the jugular foramen and its superior aspect (Figures 2A and 6). If the tumor extends to the petrous bone and petrous ICA, then a petrosal approach should be added to the operative plan [12].

Tumor vascularization should be analyzed in a pre-operative angiographic study. Branches of the ECA (occipital, ascending pharyngeal, posterior auricular, and stylomastoid foramen arteries), can be exposed in the distal carotid and stylodigastric triangles (Figure 4C). Meningeal and muscular branches of the vertebral artery can be exposed in the suboccipital and condylar triangles. Studying venous anatomy is also mandatory. The sigmoid sinus may be already occluded, and it may be possible to ligate and open it (Figures 3A and 7). The insertion of vein of Labbé should be scrutinized to decide the best area in which to perform such a ligation proximally (distal to Labbé) [9]. Venous collaterals may be enlarged and may constitute a major source of bleeding (e.g., vertebral plexus, condylar emissary vein).

Pre-operative cranial nerve deficits can dictate a more aggressive surgical approach, such as a translabyrinthine or transcochlear approach in patients with complete hearing loss (e.g., cases 13, 14, and 18); facial nerve transposition in patients with severe facial palsy; more radical resection along the lower cranial nerves in patients with vocal cord paralysis and who already underwent a tracheostomy and/or a gastrostomy tube insertion procedure (e.g., cases 17 and 18). Multidisciplinary postoperative rehabilitation of cranial nerve deficits should be anticipated and started as soon as possible to optimize the chances of long-term recovery.

## Conclusions

Despite their difficulty, lesions located in the distal cervical and postero-lateral skull base (jugular foramen and peri-condylar) regions continue to require surgical interventions in the era of stereotactic radiation. The mainstay of surgical treatment is the selection and implementation of the appropriate approach, providing adequate access without unnecessary, or even risky, dissections. Customizing the approach is simplified using anatomical compartments or triangles, which can be predicted on pre-operative imaging and unlocked in a stepwise fashion. These include the carotid and stylo-digastric triangles in the cervical region, the mastoid triangle, the jugular, condylar and sub-occipital triangles under the digastric muscle, and the deep condylar, supra-hypoglossal, and infra-hypoglossal triangles in the condylar region. The lateral distal cervical retro-auricular trans-mastoid approach is versatile and can be minimized, adjusted, or extended in each case. We provide an anatomical classification and a surgical roadmap to help young neurosurgeons plan these surgeries based on part of the senior author's (Jon H. Robertson) experience.
